# From humans to hydra: patterns of cancer across the tree of life

**DOI:** 10.1111/brv.12415

**Published:** 2018-04-16

**Authors:** Thales A. F. Albuquerque, Luisa Drummond do Val, Aoife Doherty, João Pedro de Magalhães

**Affiliations:** ^1^ Escola Superior de Ciências da Saúde, SMHN Quadra 03 conjunto A, Bloco 1 Edifício Fepecs CEP 70 710‐907 Brasilia Brazil; ^2^ Integrative Genomics of Ageing Group, Institute of Ageing and Chronic Disease University of Liverpool, William Henry Duncan Building, Room 281, 6 West Derby Street Liverpool L7 8TX U.K.

**Keywords:** comparative oncology, evolution, longevity, tumour, ageing

## Abstract

Cancer is a disease of multicellularity; it originates when cells become dysregulated due to mutations and grow out of control, invading other tissues and provoking discomfort, disability, and eventually death. Human life expectancy has greatly increased in the last two centuries, and consequently so has the incidence of cancer. However, how cancer patterns in humans compare to those of other species remains largely unknown. In this review, we search for clues about cancer and its evolutionary underpinnings across the tree of life. We discuss data from a wide range of species, drawing comparisons with humans when adequate, and interpret our findings from an evolutionary perspective. We conclude that certain cancers are uniquely common in humans, such as lung, prostate, and testicular cancer; while others are common across many species. Lymphomas appear in almost every animal analysed, including in young animals, which may be related to pathogens imposing selection on the immune system. Cancers unique to humans may be due to our modern environment or may be evolutionary accidents: random events in the evolution of our species. Finally, we find that cancer‐resistant animals such as whales and mole‐rats have evolved cellular mechanisms that help them avoid neoplasia, and we argue that there are multiple natural routes to cancer resistance.

## I. INTRODUCTION

Multicellular organisms maintain their structure and function by tightly regulating their individual cells and intercellular interactions. If this regulation fails, an abnormal tissue growth – called a neoplasm – may arise. If a neoplasm develops the ability to invade and spread to other tissues, it becomes cancer. Cancer is a major cause of death worldwide. It was the second most common cause of death in 2012 worldwide, totalling 14.1 million new cases (excluding non‐melanoma cancer of the skin) and 8.2 million deaths. As cancer is an age‐related disease, these numbers are expected to increase in future years due to population growth and aging (Stewart & Wild, 2014; American Cancer Society, 2016).

Humans are not the only species affected by cancer; in fact, only a few primitive animals and hemichordates are thought to escape the disease (Aktipis *et al.,* 2015). Furthermore, incidence rates and cancer types differ widely among species. These observations have led scientists to study other life forms to find clues on both causes and therapies for this condition. Some of these studies gave rise to animal models that help understand specific types of cancer (Schiffman & Breen, 2015), while others have focused on the general mechanisms of tumorigenesis and tumour resistance (Greaves, 2007; Finch, 2010; Caulin & Maley, 2011; DeGregori, 2011; Caulin *et al.,* 2015; Nunney & Muir, 2015). An important finding of the latter is that animals will pass on traits that grant resistance or susceptibility to cancer according to their impact on reproductive success. In other words, it is expected that cancer resistance and susceptibility is shaped by natural selection (Aktipis *et al.,* 2015).

Evidence for a role of natural selection in cancer resistance began to gain strength in 1975, when Sir Richard Peto observed that humans, despite having 1000‐fold the amount of cells and living 30 times as long as mice, had no greater chance of developing cancer. This was unexpected and unexplained because a higher number of cells and/or a higher number of cell doublings should increase the chance for mutations to occur and accumulate – it became known as ‘Peto’s paradox'. Peto suggested that mechanisms conferring resistance to cancer could have evolved in humans along with their increase in size (Peto *et al.,* 1975).

Since then, many mechanisms that enable large, long‐lived animals to survive against cancer have been discovered. For example, elephants (*Loxodonta africana*) have at least 20 copies of the tumour‐suppressor gene tumour protein 53 (*TP53*) (Abegglen *et al.,* 2015), and two species of bats of the genus *Myotis* have additional copies of the tumour‐suppressor gene F‐box protein 31 (*FBX031*) (Tollis, Schiffman & Boddy, 2017). The bowhead whale (*Balaena mysticetus*), estimated to live over 200 years, has alterations in genes associated with DNA repair, cell‐cycle regulation, and metabolic regulation (Keane *et al.,* 2015). Naked mole‐rats (*Heterocephalus glaber*) are long‐lived rodents whose high‐molecular‐mass hyaluronan is thought to mediate cancer resistance (Tian *et al.,* 2013). Such mechanisms of cancer resistance, however, are more of an exception than a rule: the goal of every organism is to reproduce, and any trait that increases fitness more than resistance to cancer does may be favoured by natural selection (Brown, Cunningham & Gatenby, 2015). Besides, as cancer is primarily caused by accumulating mutations with age (de Magalhães, 2013), it may be expected to affect all organisms with dividing cells as adults that live long enough.

Evolutionary theory posits that aging and age‐related diseases occur because of the weakening of the force of natural selection with age (Kirkwood & Austad, 2000). On one hand, detrimental genes acting late in life, such as cancer‐causing genes, will not be weeded out by natural selection. On the other hand, pleiotropic genes can increase fitness during reproductive age but also susceptibility to cancer later in life; this so‐called antagonistic pleiotropy could be responsible for the increased rate of cancer in some species, as well as for the appearance of new types of cancer (Crespi & Summers, 2006; Haig, 2015). This concept of evolutionary trade‐offs has contributed to investigations on the ties between cancer and aging: if some genes can increase reproductive success in young individuals, they might undergo positive selection even if they cause problems later in life. Strong pressure for reproductive success, combined with weak or absent pressure for resisting cancer in older age, could be the evolutionary cause of the mounting rates of cancer in aged animals, our species included (DeGregori, 2011). Moreover, there is significant variation in cancer rates across organs, which has been hypothesized to be linked to different evolutionary pressures acting on different organs, including reproductive organs (Silva *et al.,* 2011). By gaining a more complete picture of the different types of cancer affecting different animals this work will allow us to explore this hypothesis further.

There are multiple factors that influence natural selection, from slow‐changing landscapes to ever‐mutating parasites and pathogens. Therefore, it is possible that most multicellular organisms are constantly altering their susceptibility to cancer (Hochberg & Noble, 2017). To understand better how these factors influence cancer, and perhaps how cancer arises and how it may be avoided, it would be useful to juxtapose data among different multicellular organisms, or among similar organisms living under different conditions. Here, we review cancer incidence and types across the tree of life, comparing between species and with humans when the data allow, and suggesting correlations when applicable.

## II. CANCER ACROSS THE TREE OF LIFE

**Figure 1:**
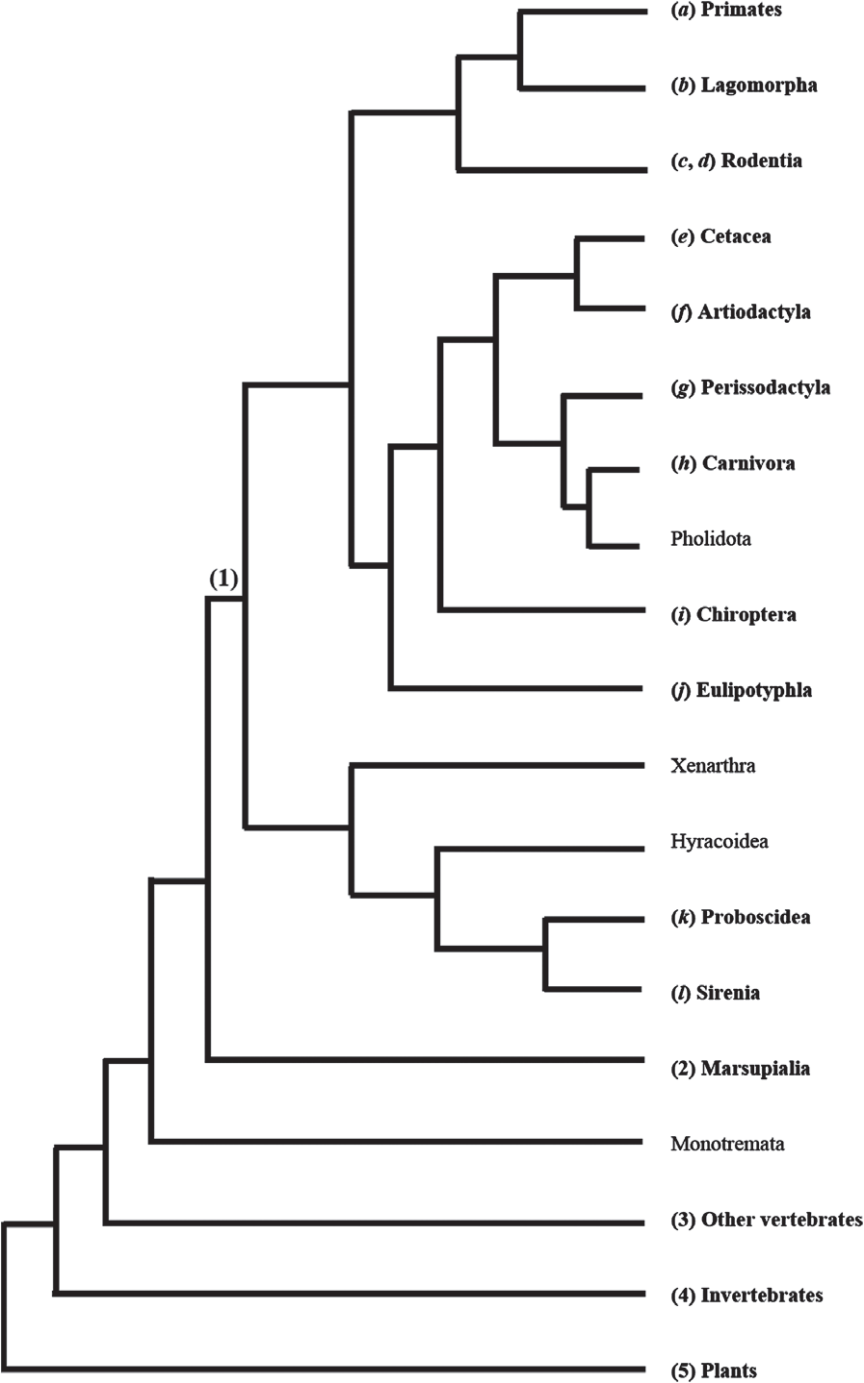
One representation of phylogeny of the mammalian orders. We found reports of cancer for all groups shown in bold. Note that we divide the Cetartiodactyla into Cetacea and Artiodactyla according to Price, Bininda‐Emonds & Gittleman (2005). Numbers and letters between parentheses next to each group indicate their section numbers in this paper.

The positions of some branches of the mammalian tree are disputed (Springer *et al.,* 2003; Kriegs *et al.,* 2006; Teeling & Hedges 2013), therefore we use Tarver *et al*. (2016) as a tool to organize our review (Fig. 1). We begin by discussing primates, then continue through the other mammalian superorders, followed by more phylogenetically distant species, non‐placental mammals, other vertebrates, invertebrates, and finally plants. As a starting point, we wrote a Perl script to search *PubMed* for >4000 species in the AnAge database using the species' scientific name plus the terms ‘neoplasia’, ‘cancer’ and ‘tumour’ (de Magalhães & Costa, 2009). A search for the total number of papers for each species without the cancer terms was also conducted as a control. The resulting hits served as basis for our survey of the literature discussed below (see Tables 1 and 2).

**Table 1:** Summary of the most common cancer types in the eutherian mammals surveyed in this study. We include only data where the most common cancers were explicitly cited or where large sample sizes allowed an estimate of the most common cancers

Taxa	Common cancer types	Environment	Reference(s)
Order Primates			
Humans	Overall population: skin carcinoma, lung cancer, colorectal cancer, breast cancer, prostate cancer. Young individuals: brain cancer and cancer in the haematopoietic system.	Modern living conditions	Stewart & Wild (2014); WONDER Online Database (2016)
Non‐human Primates	Chimpanzees: uterine leiomyoma, hepatocellular carcinoma, ovarian stromal tumours.	Captivity	Brown *et al*. (2009)
	Across all non‐human primates: gatrointestinal cancer, lymphomas, soft tissue sarcomas, breast cancer, ovary cancer, uterine cancer.	Captivity	This review; Lowenstine, McManamon & Terio (2016)
Order Lagomorpha			
Rabbits	Uterine adenocarcinoma, lymphosarcoma	Captivity (pet)	Percy, Barthold & Griffey (2016)
	Shope fibromas (virus induced), trichoblastomas (non virus‐induced)	Captivity (pet)	von Bomhard *et al*. (2007)
Order Rodentia (model organisms)			
Rats	Mammary tumour	Captivity	Greenacre (2004)
	Pituitary tumour, adrenal pheochromocytoma	Captivity	Nakazawa *et al*. (2001)
Mice	Pulmonary tumours, mammary adenocarcinoma	Captivity	Collins (1988)
	Haematopoietic tumours	Captivity	Ward (1983 **)**
Mole‐rats	Not enough data; cancer estimated to be rare	–	–
Order Rodentia (non‐model organisms)			
Hamsters	Lymphoma, adrenal cortical tumours	Captivity	Harkness & Wagner (2010)
Guinea Pigs	Bronchogenic papillary adenoma, skin cancer, cancer of the subcutis	Captivity	Kitchen, Carlton & Bickford (1975); Harkness & Wagner (2010)
Mongolian Gerbils	Female reproductive tract (especially ovary), skin cancer, cancer of the subcutis.	Captivity	Benitz & Kramer (1965); Harkness & Wagner (2010)
Black‐tailed prarie dogs	Not enough data	–	–
Chinchillas	Not enough data	–	–
Beluga whales			
Beluga whales	Intestinal cancer, epithelial neoplasms	Wild	Mikaelian *et al*. (1999); Martineau *et al*. (2002)
Bowhead whales	Not enough data; cancer estimated to be rare	–	–
Dolphins	Epithelial, lymphatic, tongue, lung, and kidney tumours	Wild	Newman & Smith (2006)
Porpoises	Not enough data	–	–
Order Artiodactyla			
Cows, pigs, and sheep	Adrenal cortex tumours	Livestock	Anderson & Sandison (1969)
	Lymphosarcomas and squamous cell carcinomas (bovines); squamous cell carcinomas (ovines); tumours of the digestive tract (swine);	Livestock	Ramos *et al*. (2008)
Order Perissodactyla			
Equines	Equine sarcoids, squamous cell carcinoma	Livestock	Ramos *et al*. (2008)
Order Carnivora			
Dogs	Soft‐tissue sarcoma, lymphoma and leukaemia, urothelial carcinoma, mammary tumour	Captivity	Schiffman & Breen (2015); Richards & Suter (2015)
Polar bears	Not enough data	–	–
Sea lions	Not enough data	–	–
Seals	Genital neoplasms, uterine leiomyomas	Wild	Bäcklin, Eriksson & Olovsson (2003); Newman & Smith (2006)
Walrus	Not enough data	–	–
Sea otters	Not enough data	–	–
Cats	Lymphoma, mammary cancer	Captivity	Vail & MacEwen (2000)
	Squamous cell carcinoma	Captivity	Zambelli (2015)
Order Chiroptera			
Bats	Not enough data; cancer estimated to be rare	–	–
Order Eulipotyphla			
African pygmy hedgehogs	Not enough data	–	–
Order Proboscidea			
Elephants	Not enough data; cancer thought to be rare	–	–
Order Sirenia			
Manatees	Not enough data	–	–

**Table 2:** Summary of the most common types of cancer in non‐mammalian vertebrates. Data are included only where the most common cancers were explicitly cited or where large sample sizes allowed an estimate of the most common cancers

Taxa	Common cancer types	Environment	Reference(s)
Other vertebrates				
Birds	Cancer of the integument, urinary system, and genital system	Captivity (pet)	Filippich (2004)	
	Lipoma, lymphosarcoma, fibrosarcoma (Amazon parrots)	Wild	Levine & Practice (2003)	
Reptilians	Liver tumour (necropsied snakes and lizards) Skin tumour (live snakes)	Captivity (zoo)	Sykes & Trupkiewicz (2006)
	Epithelial neoplasm (snakes)	Captivity (zoo)	Ramsay *et al*. (1996)
Amphibians	Skin tumours	Unknown	Balls (1962)
Fishes	Papilloma, malignant tumours of the connective tissue proper, melanomas	Unknown	Schlumberger & Lucké (1948)

### (1) Eutherians (placental mammals)

#### (a) *Order Primates*

##### (i) *Humans*

Cancer rates among humans are striking, and they are bound to keep increasing. We still do not know all the mechanisms that lead to such a high incidence of cancer in our species, but one hypothesis is that cultural changes and technological advances have produced the greatest of evolutionary mismatches – a situation where the environment changes into something different from that which an inhabiting species is adapted to, and that provokes stress and may increase susceptibility to cancer, among other problems (Hochberg & Noble, 2017). Indeed, the only animals that have cancer rates anywhere near those of humans are those subjected to similar life conditions, or those whose habitat gets contaminated by sub‐products of human lifestyles. Our increasing life expectancy allows the appearance of cancers that would not have affected our ancestors. These types of cancer would have emerged only in later life, or if emerging in early life they would not have affected fitness enough to cause strong selection for resistant individuals (de Magalhães, 2013).

The most common cancer type in humans is carcinomas of the skin, particularly squamous cell carcinoma and basal cell carcinoma. However, these two types of carcinoma, also referred to as keratinocyte carcinoma (KC), are generally not reported because they hardly ever produce metastases (von Domarus & Stevens, 1984). Skin colour is a defence against KC. Greaves (2014) has hypothesized that skin cancer could have been a potent selective force for the emergence of black skin in *Homo sapiens*, and that pale skin reflects an adaptive shift that happened when humans migrated into Europe 50000–80000 years ago. Indeed, genes associated with skin pigmentation are known to be divergent between Africans and Eurasians (de Magalhães & Matsuda, 2012). On the other hand, Jablonski & Chaplin (2014) argue that early humans had inherited the capacity for heavy tanning from their ape ancestors, and that stronger evolutionary pressure than skin cancer would be necessary to select for the hairless, permanently dark skin found in some populations. Regardless of which side is right, it makes sense for cancers with low impact on fitness such as KC to be those with the highest incidence.

The second most common type of cancer in humans is that of the lung, which represented 1824701 (16.7%) of the 14.1 million new cases of non‐KC reported in 2012. Lung cancer is perhaps the one in which environmental influences are best understood. Tobacco smoke contains more than 7000 chemical compounds, many of which are carcinogens. Smokeless tobacco products contain 3000 chemicals, many of which are also carcinogens (Stewart & Wild, 2014). As with most cancers, however, there are also genetic factors involved in this disease (Brennan, Hainaut & Boffetta, 2011). Smoking is also a risk factor for other cancers, including oesophageal (3.2% of non‐KC cancer incidence in 2012), and bladder cancer (3.1% of non‐KC cancer incidence in 2012). However, these cancers have other risk factors of their own, such as ingestion of alcohol and hot beverages for oesophageal cancer and chronic infection with *Schistosoma haematobium* for bladder cancer (Stewart & Wild, 2014).

**Figure 2:**
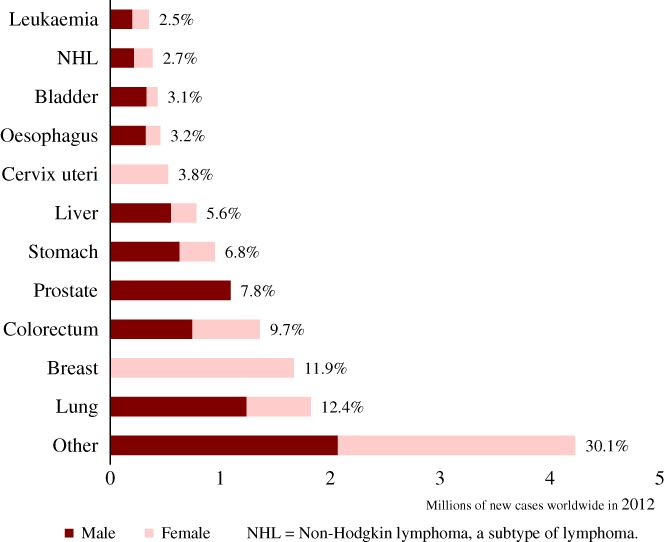
New cases of cancer worldwide in 2012 (crude values). Percentages show the contributions of each type of cancer to the overall number of new cases combining both sexes. Created using data from GLOBOCAN (Ferlay et al., 2013).

Breast cancer affects mainly females, and is the third most common cancer among humans (11.9%; see Fig. 2). Hormonal status influences the incidence of this type of cancer, and risk factors include total age, use of oral contraceptives, hormone replacement, age at menarche and menopause, age at first pregnancy and nulliparity, a previous case of breast cancer, and family history (McPherson, Steel & Dixon, 2000). A study found that multiparity was protective against breast cancer (Hinkula *et al.,* 2001), so it could be that the low birth rate associated with the modern lifestyle is potentiating the incidence of this disease. Diet also plays an important role, especially the consumption of fats and compounds with oestrogenic activity, the so‐called xenoestrogens. The impact of environmental factors is reinforced by the low rate of inherited breast cancer and by the high incidence in countries that adopt western diets.

Breast cancer affects males at the rate of 1% of all cases of this disease. For unknown reasons, this ratio is higher among black populations (Sasco, Lowenfels & Paskerdejong, 1993).

Colorectal cancer is the fourth most common worldwide (9.7%). As cells of the colon are in direct contact with gastrointestinal content, ingested carcinogens may influence the development of cancer. One well‐studied group of food carcinogens are heterocyclic amines, which are produced when heating amino acids and sugar present in meat (Sugimura, 2000). This could explain why approximately 44% of colon cancer cases are concentrated in countries where the diets are high in meat products (Stewart & Wild, 2014). Dietary carcinogens such as nitrosamines are also implicated as important causes of gastric carcinomas (Tsugane & Sasazuki, 2007).

Prostate cancer is the fifth most common type of cancer worldwide (7.8%), and the third most common among males (15.0%) (Stewart & Wild, 2014). As with breast cancer, prostate cancer is affected by hormonal and dietary factors and its incidence increases after reproductive age. Despite having lower incidence rates than lung cancer, breast and prostate cancers have lower mortality and together are more prevalent in the population.

Cancer also varies widely across different age groups. In the USA, leukaemia accounts for 32.1% of all cancer in children aged 1 to 14 years, followed by cancers of the brain and other nervous system (22.6%), lymphoma (10.3%), and soft tissue sarcomas (5.5%) (WONDER Online Database, 2016).

**Figure 3:**
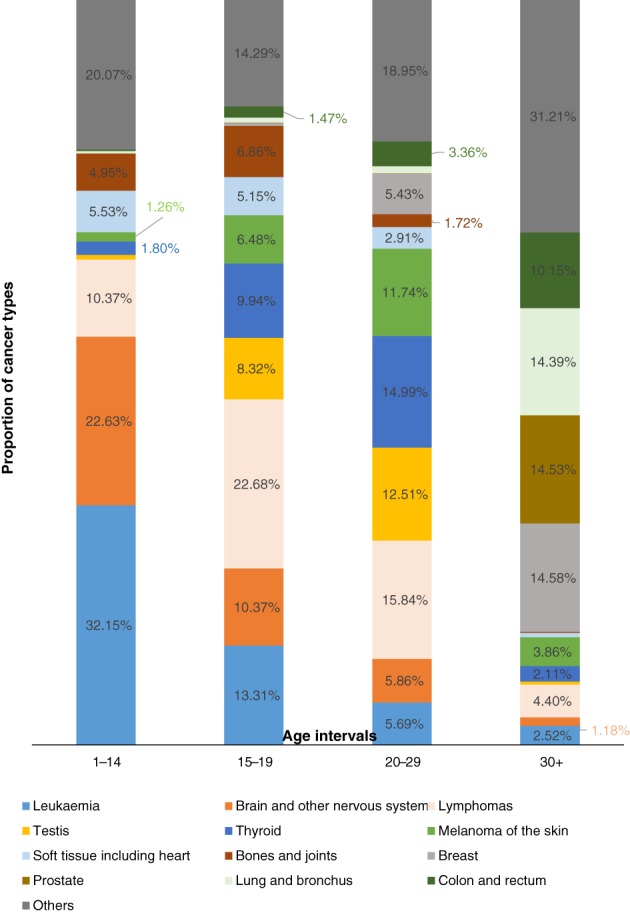
Changes in the cancer proportions from childhood to early adulthood; only rates above 1% are shown. Created using data from the Centers of Disease Control and Prevention's WONDER Online Database (2016).

In adolescents aged 15 to 19 years, lymphomas are the most common cancer types (22.6%), followed by leukaemia (13.3%), brain and nervous system tumours (10.3%), thyroid carcinoma (9.94%), and testicular tumours (8.32%) (WONDER Online Database, 2016). Figure 3 shows that as individuals enter adulthood, the proportion of brain tumours, leukaemia, and lymphoma decrease, while that of melanoma, testicular cancer, and thyroid cancer increase.

This age‐dependent pattern of cancer incidence is important because natural selection should act against such fitness‐damaging cancers during pre‐reproductive and reproductive ages (Leroi, Koufopanou & Burt, 2003; Nunney & Muir, 2015). One hypothesis for the high proportion of haematopoietic tumours in the young is that the high mutability of some pathogens leads to an arms race between host and parasite that exerts constant evolutionary pressure on both sides – the host's immune system is constantly selected for new protective traits, and the pathogens mutate to overcome these traits. Such traits, however, may have the side effect of facilitating the appearance of tumours. However, an explanation for childhood leukaemia may be more complex, as suggested by the delayed infection hypothesis, which considers the excessive protection of young children as a cause for an evolutionary mismatch in their immune system, leading to aberrant responses in the immune system that may contribute to cancer (Greaves, 2006). An alternative hypothesis is that cells of the haematopoietic system evolve differently in adults and children; although selection plays a large role in the evolution of these cells in the former, genetic drift is more important in the latter. There is a possibility that other common childhood cancers appear due to genetic drift, and more research in this area would be beneficial (Rozhok, Salstrom & DeGregori, 2016). As for tumours of the nervous system, one cause could be attributed to the recent evolution of larger brains in our species; strong selection on a highly adaptive trait like brain size could bring along costs, such as increased cancer risk (Leroi *et al.,* 2003).

The proportion of testicular cancer reaches its peak between 30 and 40 years of age, after which it decreases. This high incidence of testicular cancer (Fig. 3) in young men still lacks an explanation, although it could also be related to how selection on one trait can lead to associated costs in terms of increased cancer risk. Indeed, human reproductive organs tend to have a higher mass‐normalized cancer incidence (Silva *et al.,* 2011). In the case of testicular cancer, one possible cause could be the elevated activity of reproductive tissue during puberty and early adulthood (de Magalhães, 2013). Genetics may also be involved: studies show positive selection for several genes implicated in both male reproductive fitness and cancer (Crespi & Summers, 2006). Another possible explanation could be that testicular cancer is highly influenced by environmental factors: in most developing countries, the age‐standardized rates are below 3 per 100000, while in most developed countries it is above that number. In the USA, the rates are 5 per 100000; in the UK, 6.8; and Norway, where they are highest, 12.2. There also seems to be a correlation between western/eastern cultures and testicular cancer. In Japan, the age‐standardized rates are 2.1; in China, they are 0.3 (Ferlay *et al.,* 2013).

Some hypotheses for the disparity in incidence among countries point out that in developed regions there has been an increase in predisposing factors such as cryptorchidism (undescended testes), hypospadias (a misdeveloped urethra that opens at the base of the penis), and infertility (Bray *et al.,* 2006; Cancer Research UK, 2016). The reason behind increases in these factors, however, remains a mystery. One well‐known possibility is that excessive exposure to oestrogen during the foetal stage could increase the risk for such abnormalities (Skakkebék *et al.,* 1998). The data are still inconclusive, and more studies are necessary to understand better the aetiology of testicular cancer.

Other cancers beside testicular vary in incidence among countries. In regions of high Human Development Index (HDI), there is a higher incidence of breast, prostate, lung, and colorectal cancer, while in regions of low HDI there is a higher incidence of infection‐related cancers, such as those of the liver and of the uterine cervix. Examining differences in cancer among humans living under different social status may provide important clues about the aetiology of different types of the disease (Stewart & Wild, 2014).

##### (ii) *Non‐human primates*

Puente *et al*. (2006) showed that every human cancer gene has an orthologue in non‐human primates, and genomic analyses in these animals have improved our understanding of the evolution of some tumour‐suppressor genes. *S*tudies in monkeys have also helped advance our understanding of chemical carcinogens, including the tumour‐inducing effect of some anti‐neoplastic agents and that of exogenous oestrogens (Xia & Chen, 2011).

Scientists previously thought that non‐human primates rarely developed cancer, but recent studies suggest that tumours might occur frequently in this group (Lapin & Yakovleva, 2014). Brown *et al*. (2009) found 102 naturally occurring and 12 experimentally induced neoplasms among chimpanzees (*Pan* spp.). The most common of these were uterine leiomyomas, followed by hepatocellular carcinomas and ovarian stromal tumours.

**Figure 4:**
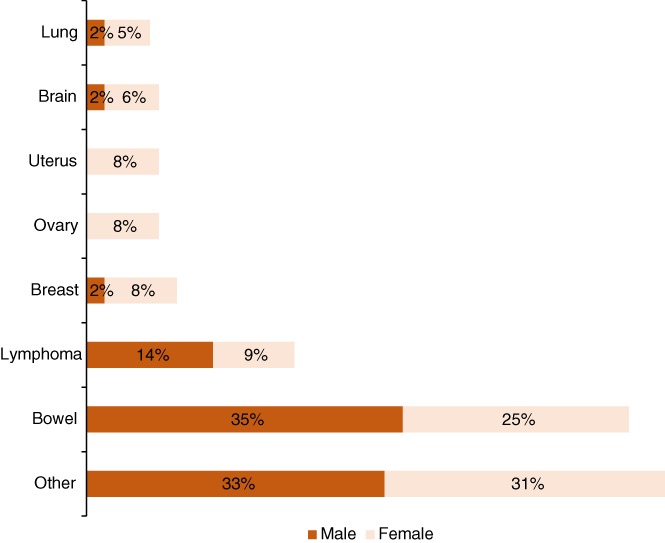
Proportion of different types of cancer in male and female non‐human primates. Created using our own data derived from a survey of the literature.

Based on our literature search (Fig. 4), we found cancer cases across 29 non‐human primate species; we observed that the most common type of cancer in non‐human primates of both sexes was bowel cancers, followed by lymphomas and soft tissue sarcomas in males and by lymphomas, breast, ovary, and uterus cancer in females. Among the species studied, cotton‐top tamarins (*Saguinus oedipus*) were one of the most prone to developing colorectal carcinomas. Liu & Russell (2008) hypothesized that this could be due to unbalanced nutrition, intake of refined food, and the susceptibility of these animals to stress. Crab‐eating macaques (*Macaca fascicularis*) and rhesus monkeys (*Macaca mulatta*) were also among the groups with high incidence of bowel cancer (Valverde *et al.,* 2000).

Lymphomas were the second most common type of cancer among the 29 species studied. Although the reported cases comprised spontaneous tumours, lymphomas in non‐human primates usually have viral aetiology (Holmberg *et al.,* 1985; Hofmann *et al.,* 2001; Cianciolo *et al.,* 2007; Tardif *et al.,* 2011; Gibson‐Corley & Haynes, 2012).

A review by Lowenstine *et al*. (2016) also shows a high incidence of lymphomas among non‐human primates, which as we have discussed is common in young humans, particularly in those in the age range of 1 to 19 years. This adds to the suggestion that cancers of the immune system are common among multicellular organisms in both controlled environments and the wild.

Breast carcinoma, despite representing a large percentage of the cancer types in non‐human primates, occurs more rarely in this group than in human females. However, some of the groups underwent interventions that could have been protective against breast cancer: release from captivity before postmenopausal age, multiparity and ovary removal, and feeding with a balanced diet rich in soy protein (Wood *et al.,* 2006).

The high incidence of bowel cancers and cancers of the female reproductive tract in captive non‐human primates also suggests that certain habits influence their occurrence. The already‐mentioned high intake of refined food and unbalanced diets could be analogous to the dietary changes in humans, and their susceptibility to stress could be related to our own changes in behaviour due to societal pressure.

It is important to note that some cancers that are common in humans are rare in non‐human primates. We are only aware of one case of lung cancer (Cho *et al.,* 2007), possibly due to the absence of carcinogenic tobacco smoke. Testicular and prostate cancer seem to be rare as well: we found only a few reports of seminomas and one report of a Leydig cell tumour (McClure, 1973; Brack, 1988; Gozalo, Nolan & Montoya, 1992). These types of cancer are among the most common in human males (testicular cancer in younger males and prostate cancer in older males). As previously mentioned, the higher rates for testicular cancer in humans are still under investigation. Prostate cancer, on the other hand, is known to be influenced by aging: humans have also evolved a much longer lifespan than non‐human primates, which possibly increases their chances for developing the disease (Finch, 2010).

Finally, our data show that the age of onset of cancer in non‐human primates is ∼3 years old or more, which corresponds to the beginning of adulthood; moreover, Lowenstine *et al*. (2016) found that cancer mortality in chimpanzees increases from ages under 35 years old (8.3%) to ages above 35 years old (9.0%). These findings suggest a correlation between lifespan and the onset of cancer.

#### (b) *Order Lagomorpha*

##### (i) *Rabbits*

The most common tumours in European rabbits (*Oryctolagus cuniculus*) are uterine adenocarcinomas followed by lymphosarcomas (Percy *et al.,* 2016). The incidence of lymphomas varies across continents. They are rare in North American pet rabbits, but common in European pet rabbits (Ritter *et al.,* 2012). Researchers found no indication of a viral aetiology (Ritter *et al.,* 2012).

Mammary cancers are rarely reported in laboratory and pet rabbits. A retrospective study encompassing all mammary tumours and tumour‐like lesions diagnosed in pet rabbits between July 2004 and September 2011 found that the most common tumours of the mammary gland were adenocarcinoma and invasive carcinoma (Schöniger, Horn & Schoon, 2014).

von Bomhard *et al*. (2007) conducted a retrospective study to examine cutaneous neoplasms from 179 pet rabbits. The authors divided tumours into virus‐induced and non‐virus‐induced. The most common virus‐induced tumours were Shope fibromas, which affected 19/179 rabbits, and the most common non‐virus‐induced tumours were trichoblastomas, which affected 58/179 rabbits. They also investigated mesenchymal tumours, of which the most common were lipomas, myxosarcomas, and fibrosarcomas. Among the 179 rabbits, there were also eight cases of malignant melanoma.

#### (c) *Order Rodentia (model organisms)*

Studies with model rodents have contributed greatly to the understanding of cancer in humans. For example, tumour transplantation experiments have shown that growth of latent tumours declines with age, but their survivability increases, which is compatible with current data on humans; exogenous oestrogen administration is carcinogenic in both humans and mice, and the difference in cancer incidence between mice of different sexes corresponds to that in humans, a phenomenon that deserves further investigation (Anisimov, Ukraintseva & Yashin, 2005). On the other hand, some characteristics are unique to rodent tumours: it is common for spontaneous tumours to regress in rodents, but not in humans, and many human carcinogens are not carcinogenic in rodents (Anisimov *et al.,* 2005). Although data from rodents cannot always be extrapolated to humans, they remain an important cancer model.

##### (i) *Rats*

The literature reports a tumour incidence of up to 87% in rats. The incidence and types of tumour vary depending on many factors, such as the strain of the rat, age, sex, diet, caloric intake and environment (Greenacre, 2004). There is controversy regarding the most common tumours in this species. Some studies suggest they are those of the mammary gland (Greenacre, 2004), but others have found that the most common were pituitary adenoma and adrenal pheochromocytoma when considering both sexes, testicular interstitial cell tumours when considering only males, and mammary gland tumours, thyroid C‐cell adenomas, and uterine stromal polyps when considering only females (Nakazawa *et al.,* 2001). Other common types of tumour in rats include mononuclear cell leukaemias, malignant lymphomas (Greenacre, 2004), spinal cord tumours and renal tumours (Harkness & Wagner, 2010).

##### (ii) *Mice*

Tumourigenesis has been extensively studied in mice; there are strains of mice developed with specific susceptibilities and resistances to certain tumours (Greenacre, 2004). Similar to rats, there is controversy as to the most commonly occurring tumour types, and cancer susceptibility appears to be strain‐dependent (Sibilia & Wagner, 1995; Woodworth *et al.,* 2004). Some sources suggest that pulmonary tumours are among the most common, while others suggest that mammary adenocarcinoma is the most common tumour, at least in aged female mice, contributing from 30 to 70% of total cancer burden (Collins, 1988). Two other sources show haematopoietic tumours as the most common in these animals (Ward, 1983; Frith, Ward & Chandra, 1993). Also common are hepatocellular carcinomas, lymphocytic leukaemias (Greenacre, 2004), testicular interstitial cell tumours and ovarian tumours (Harkness & Wagner, 2010). Interestingly, even long‐lived wild‐derived mice die primarily of cancer (Harper, Leathers & Austad, 2006).

##### (iii) *Mole‐rats*

The naked mole‐rat is a long‐lived rodent native to East Africa. It exhibits extraordinary resistance to cancer and has been studied in order to understand how such resistance is generated. It can live up to 30 years, whereas the longevity of similarly sized mice is 2–3 years (Austad, 2010). Scientists have investigated these animals and identified key genomic variations that may have contributed to the evolution of longevity and resistance to cancer (Keane *et al.,* 2014; Davies *et al.,* 2015). Despite these findings, Delaney *et al*. (2016) found tumours in two zoo‐housed naked mole‐rats. Taylor, Milone & Rodriguez (2016) followed with a report on four cases of spontaneous neoplasia in naked mole‐rats pertaining to the exhibit collection at Disney's Animal Kingdom. The examined population descended from a single pair, which could have facilitated tumour‐driving mutations. On the other hand, naked mole‐rats inbreed even in the wild, suggesting that the increased cancer rate in this study might have other causes. Another issue is that with a lifespan of over 30 years, old naked mole‐rats are relatively rare in captivity, and therefore we may observe more cancer cases as older animals are examined.

Researchers have found that high‐molecular‐mass hyaluronan is involved in the arresting of cell growth once cells come into contact with extracellular matrix (Seluanov *et al.,* 2009) and also the activation of a modified version of the inhibitors of cyclin‐dependent kinase 4 (*INK4a*) locus, which contains tumour‐suppressor genes (Tian *et al.,* 2015). Faulkes *et al*. (2015) found that high‐molecular‐mass hyaluronan is generated from two different mutations in the hyaluronan synthase 2 gene (*HAS2*), one of which is shared among all African mole‐rats.

A second group of mole‐rats has shown remarkable cancer resistance. The mole‐rats of the genus *Spalax*, also known as blind mole‐rats, can avoid not only spontaneous tumours, but induced tumours as well (Manov *et al.,* 2013). They, too, produce high‐molecular‐mass hyaluronan, but they have neither of the two mutations in the *HAS2* gene previously identified as key for the production of the modified molecule, which means they may have other mechanisms of producing this molecule (Faulkes *et al.,* 2015). Additionally, blind mole‐rats resist cancer by at least one other mechanism: the release of interferon‐β (IFN‐β) in the face of excessive proliferation, which then activates tumour‐suppressor pathways (Gorbunova *et al.,* 2012).

As with elephants, the reproductive fitness of naked mole‐rats increases with age, which adds strength to the hypothesis that this could contribute to the selection of cancer‐resistance traits (Brown & Aktipis, 2015).

#### (d) *Order Rodentia (non‐model organsims)*

##### (i) *Hamsters*

Cancer has been reported in at least three different types of hamsters: Syrian (*Mesocricetus auratus*), Chinese (*Cricetulus griseus*), and Russian hamsters (*Phodopus sungorus*). Syrian hamsters have a tumour incidence of 3.7%, and high susceptibility to induction of a wide variety of tumours with carcinogenic agents, which makes them ideal for carcinogenic studies (Greenacre, 2004). Age is an important factor in cancer incidence in hamsters; reports show an incidence of up to 50% in hamsters greater than 2 years of age (Kikman & Algard, 1968; Strandberg, 1987).

There is debate as to what is the most common type of tumour in hamsters. While older studies suggest it to be lymphoma, newer research shows that adrenal cortical tumours are the most common. This controversy may be due to fewer hamsters being currently exposed to a virus known to cause lymphoma. Other common tumours in hamsters are those of the skin and subcutis and those of the gastrointestinal system (Harkness & Wagner, 2010).

##### (ii) *Guinea pigs*

Tumours are uncommon in guinea pigs (*Cavia porcellus*), and their incidence varies among strains. There is evidence correlating cancer incidence with aging, and neoplasia in animals aged >3 years old has been reported to be up to 30% (Greenacre, 2004). Bronchogenic papillary adenoma is the most commonly reported tumour, ranging from 30 to 35% of all neoplasms in guinea pigs more than 3 years old (Kitchen *et al.,* 1975; Collins, 1988; Cooper, 1994; Harkness & Wagner, 2010). The next most common are tumours of the skin and subcutis (Greenacre, 2004), which comprise approximately 15% of all neoplasms in guinea pigs (Harkness & Wagner, 2010).

##### (iii) *Mongolian gerbils*

Tumour incidence in gerbils (*Meriones unguiculatus*) varies from 8.4 to 26.5% (Benitz & Kramer, 1965; Vincent, Rodrick & Sodeman, 1979; Harkness & Wagner, 2010), the most common type being those of the female reproductive tract, specifically of the ovary (Collins, 1988; Harkness & Wagner, 2010), which was found to occur in 12.5% of female gerbils (Benitz & Kramer, 1965). The second most common type is that of the skin and subcutis, mainly of the ventral abdominal scent gland (Greenacre, 2004).

There are also reports of lymphoreticular tumours in gerbils, including Hodgkin's‐like lymphoma and lymphoblastic lymphoma (Rembert *et al.,* 2000). Surprisingly, we found no reports of mammary tumours in gerbils.

##### (iv) *Black‐tailed prairie dogs and chinchillas*

There are few publications regarding tumours in prairie dogs (*Cynomys ludovicianus*) and chinchillas (*Chinchilla lanigera*). A report on two prairie dogs shows hepatocellular carcinomas with metastasis to the lung (Une *et al.,* 1996); the researchers found no traces of a viral infection (Phalen, Antinoff & Fricke, 2000).

In chinchillas, one report revealed a case of uterine leiomyosarcoma in a 1‐year‐old female (Mans & Donnelly, 2012). There have also been reports of malignant lymphoma, lymphosarcoma, gastric adenocarcinoma (Lucena *et al.,* 2012), and adenocarcinoma of the lung (Greenacre, 2004).

#### (e) *Order Cetacea*

##### (i) *Beluga whales*

A number of studies have focused on Beluga whales (*Delphinapterus leucas*) from the St. Lawrence estuary. Data over 17 years from this group showed an estimated annual cancer rate of 163/100000, of which 63/100000 were of intestinal origin (Martineau *et al.,* 2002).

The contaminants in the St. Lawrence River estuary are thought to increase carcinogenesis in beluga whales living there compared with animals living elsewhere. A study analysed 50 carcasses of Arctic beluga whales and found no occurrence of neoplasia (De Guise, Lagacé & Béland, 1994), but since the cancer rates in the estuary were approximately 1/600, there is not enough evidence to conclude whether the contaminants are the culprit.

Epithelial neoplasms are common in beluga species, (Mikaelian *et al.,* 1999; Newman & Smith, 2006). Many of its subtypes may be associated with chemical carcinogens present in the estuary (De Guise *et al.,* 1994).

##### (ii) *Bowhead whales*

The bowhead whale has been estimated to live over 200 years, and there are no reported cases of cancer in this species. Given their large size, their cells must have a lower chance of developing into cancer when compared to human cells, possibly due to protective molecular mechanisms (de Magalhães, 2015; Keane *et al.,* 2015). Cancer in large whale species could go undetected, however, because stranded animals are rare and we know very little about their natural causes of death. Bowhead whales are also harvested by native peoples, but it is typically the young adults who are taken.

##### (iii) *Dolphins and porpoises*

There are more reports of neoplasia in dolphins than in other marine mammals, especially epithelial tumours, followed by those of the lymphatic system, tongue, lung, and kidney (Newman & Smith, 2006). Among the reports we found, testicular tumours and T‐cell lymphoma are noteworthy for also being common in young humans (Mawdesley‐Thomas, 1975; Migaki, Woodard & Goldston, 1978; Newman & Smith, 2006; Arbelo *et al.,* 2014). There are rare reports of neoplasia among porpoises, one of which includes a renal teratoma (Baker & Martin, 1992).

##### (iv) *Other cetaceans*

There are few reports of neoplasia among other cetaceans. One study examined 55 pilot whales (*Globicephala melaena*) from Newfoundland and encountered concomitant uterine leiomyoma and vaginal fibroleiomyoma in a single animal (Cowan, 1966). Another study examined 2000 whales from a South African whaling expedition and found benign tumours in a sperm whale (*Physeter macrocephalus*) and in a sei whale (*Balaenoptera borealis*) (Newman & Smith, 2006).

#### (f) *Order Artiodactyla*

##### (i) *Cows, pigs and sheep*

Reports on these animals are rare, especially for those living in the wild. Thus, animals kept as livestock are particularly valuable in furthering our understanding of cancer patterns in Artiodactyla.

A histological survey collected specimens in 100 UK abattoirs and found 302 tumours in 1.3 million cattle, 107 in 4.5 million sheep, and 139 in 3.7 million pigs (Anderson & Sandison, 1969). The objective was to examine tumours of the endocrine glands, with exception of the ovary and brain. The most common tumours found were those of the adrenal cortex. Other tumours included adrenal carcinomas, pheochromocytomas, thyroid carcinomas, and one islet‐cell carcinoma (in a pig). The authors found no aetiology for these cancers, with exception of thyroid carcinomas, which had goitre as a risk factor. As in humans, goitre was related to a deficiency of iodine. The authors suggested that the tumour profile might have been skewed due to the early killing of these animals in the abattoirs (Anderson & Sandison, 1969).

A retrospective study of pancreatic tumours in cattle identified 16 primary tumours, 11 of which were islet cell tumours. As in humans, these tumours produced the same hormones as normal tissue. Unlike humans, they presented with either endocrine or exocrine cells (whereas in humans a single tumour presents with both types of cells). Amyloid is present in pancreatic tumours of many species, but it was not present in tumours collected in this study (Kelley, Harmon & McCaskey, 1996).

A second retrospective study comprised all cases received by the veterinary department of Universidade Federal de Pelotas, Brazil. The researchers analysed 6267 specimens and found 175 tumours, distributed in the following manner: 98/4407 in bovines (2.22% incidence), 9/636 in ovines (1.41% incidence), 65/774 in equines (8.39% incidence), and 3/450 in swine (0.6% incidence). The authors suggested that the lower incidence in swine and ovines compared to bovines and equines could be due to swine and ovines being put down at younger ages (Ramos *et al.,* 2008).

The most common tumours in bovines were lymphosarcomas, followed by squamous cell carcinomas and fibromas. Lymphomas in bovines are correlated with bovine leukaemia virus and with herd size. The authors suggested this last factor could imply a genetic influence in the development of this type of cancer (Ramos *et al.,* 2008). In ovines, the most common tumours were squamous cell carcinomas. Two of the three tumours identified in swines were of the digestive tract – one gastric adenoma and one oral cavity fibroma – and the other was a melanoma (Ramos *et al.,* 2008).

#### (g) *Order Perissodactyla*

##### (i) *Horses*

Ramos *et al*. (2008) found that the most common tumours in equines were equine sarcoids, followed by squamous cell carcinomas. Only seven of the 65 equine tumours were of haematopoietic origin, but research has suggested immunohistochemical similarities between T‐cell‐rich, large B‐cell lymphomas of equines and humans. Studies have also found viral RNA in these tumours, although they were unable to identify these viruses as the cause (Kelley & Mahaffey, 1998).

#### (h) *Order Carnivora*

##### (i) *Dogs*

In the USA, over 4.2 million dogs (∼5300 in 100000) develop cancer every year, compared to 1.66 million humans (∼500 in 100000) (Schiffman & Breen, 2015), although of course the higher risk of cancer for dogs per year is expected given their shorter lifespan (5–10 years). Dogs are one of the most explored non‐rodent models in comparative oncology for a number of reasons. For example, their sporadic tumours resemble those of humans. Dogs are kept in protective household environments and over 40 billion dollars is spent annually on dog health care, a level that is second only to humans (Rowell, Mccarthy & Alvarez, 2012). Finally, inbreeding practices that aim to select certain traits have reduced their genetic variability and increased susceptibility to different types of cancer across different races, which facilitates the isolation of genes involved in cancer and subsequent comparisons with humans (Rowell *et al.,* 2012; Schiffman & Breen, 2015).

Findings from the Animal Tumor Registry of Genoa, Italy, show that cancer incidence is proportional to age in dogs, with 28% of the tumours occurring in those older than 11 years old and 3.9% occurring in those younger than 3 years old (Merlo *et al.,* 2008). The most common types of malignant cancers developed in dogs are lymphoma, mast cell tumour, osteosarcoma, soft tissue sarcoma, mammary carcinoma (Dobson *et al.,* 2002; Bronden *et al.,* 2010), oral melanoma and haemangiosarcoma (Maekawa *et al.,* 2016). Dogs are also the only known species, apart from humans, to significantly develop spontaneous prostate cancer (Greaves, 2007).

A number of cancers have a low incidence in humans, and a higher incidence in dogs (Schiffman & Breen, 2015). For example, soft‐tissue sarcomas form approximately 1% of new cases of cancer in humans. However, such cancers occur approximately 75 times more frequently in dogs, with some breeds being more susceptible than others (Rowell *et al.,* 2012). Comparisons between canine and human osteosarcoma have allowed advancements such as identification of several subtypes (Scott *et al.,* 2011), driver mutations (Angstadt *et al.,* 2012), and biomarkers (Rankin *et al.,* 2012; Ren & Khanna, 2014), and have led to new therapeutic investigations (Rankin *et al.,* 2012; Yin *et al.,* 2016).

Lymphomas and leukaemias are common in both humans and dogs. In 2014, there were 250000 estimated cases of canine lymphoma compared to 71000 human diagnosed cases of non‐Hodgkin lymphoma (which is comparable to canine lymphoma). Lymphomas in dogs are similar to those in humans in molecular aspects and in their response to therapy; canine studies have been and will continue to be important in the comprehension of this disease (Richards & Suter, 2015). Genomic studies of canine leukaemias are still underway, but recent results show similarities between canine and human leukaemias. Glioblastomas and melanomas also show genomic similarities (Schiffman & Breen, 2015).

Urothelial carcinomas are also common in both humans and dogs. However, their occurrence in humans seems to be more due to environmental factors, such as cigarette smoke, while in dogs they seem to occur due to genetic predisposition (Schiffman & Breen, 2015).

In female dogs, mammary gland tumours represent 70% of all cancer cases (Merlo *et al.,* 2008). Mammary adenocarcinoma is a common subtype and dogs with these tumours often suffer recurrences or metastases if treated by surgery alone (Merlo *et al.,* 2008). Hormones involved in human mammary carcinogenesis, such as oestrogen, progesterone, prolactin, growth hormone (GH) and insulin‐like growth factor 1 (IGF‐1) have also been associated with canine mammary carcinogenesis (Queiroga *et al.,* 2011). Histological patterns of breast cancer are similar among humans and canines, but cytological patterns differ (de las Mulas & Reymundo, 2000).

Finally, it is worth noting that there are many genetic similarities between canine and human cancer: scientists found several overlapping loci between dogs and humans with osteosarcoma (Rowell *et al.,* 2012; Shearin *et al.,* 2012), and tumour markers for many cancers are similar in both species (Queiroga *et al.,* 2011; Pinho *et al.,* 2012).

As with all animals, there are limitations to the usefulness of canines as a comparative cancer model (Mayeux, 2004; Farrell *et al.,* 2015). It is also important to consider that our data from dogs come from domestic animals or animals kept in captivity. Nevertheless, the findings from the present section indicate that studies in dogs are likely to help us understand the pathogenesis of cancer in humans.

##### (ii) *Polar bears*

There are reports of two polar bears (*Ursus maritimus*) developing hepatic neoplasia: one hepatocellular carcinoma and one cholangiocarcinoma. The exact aetiology is unknown, but it is thought to involve exposure to industrial chemicals, clonorchid flukes, and viral agents (Newman & Smith, 2006). Another hypothesis suggests that transition from intermittent feeding in the wild to continual feeding in the zoo environment could have a role in the process (Newman & Smith, 2006).

A 25‐year‐old polar bear presented with pancreatic islet‐cell tumour (Alroy, Baldwin Jr & Maschgan, 1980), and here also the authors suspected there could be an influence of dietary factors. In this case, it was suggested that high‐carbohydrate diets could have been the predisposing factor. A 7‐year‐old polar bear was reported to have developed osteosarcoma (Ponomar'kov & Khutorianskiĭ, 1995).

##### (iii) *Sea lions*

Brown *et al*. (1980) reported metastatic adenocarcinoma in a female and in a male California sea lion (*Zalophus californianus*). These authors suspected the tumours to have originated in the urogenital tract. Another study reported widespread metastases of a poorly differentiated squamous cell carcinoma in a California sea lion (Joseph, Cornell & Migaki, 1986). Gulland *et al*. (1996) examined 370 California sea lions and found 66 of them (18%) had similar metastases, which they suspected to have originated from transitional‐cell carcinoma.

In another study, researchers found an association between urogenital carcinoma and otarine herpesvirus‐1 – this virus is closely related to human herpesvirus 8, which is implicated in Kaposi's sarcoma in humans (Lipscomb *et al.,* 2000). There have also been reports on several types of malignant epithelial carcinomas, but it appears that, like some of the cases previously discussed, most may have originated from genital‐tract metastases (Newman & Smith, 2006).

A single California sea lion was diagnosed with both a simple mammary carcinoma and a complex mammary carcinoma (Matsuda *et al.,* 2003). Complex mammary carcinomas develop in other species such as dogs, but not in humans.

##### (iv) *Seals*

The most reported neoplasms in seals were those of the genital tract (Newman & Smith, 2006). A study reported 64% incidence of uterine leiomyomas in aged Baltic gray seals (*Halichoerus grypus*) (Bäcklin *et al.,* 2003). The authors suggested that organochlorines with hormone‐like properties could have overstimulated smooth‐muscle proliferation, since seals' tissues had high levels of dichlorophenyl trichloroethane (DDT) and polychlorinated biphenyls (PCBs).

Other types of cancer, such ovarian granulosa cell tumour and lymphosarcoma, have also been reported (Newman & Smith, 2006). Additionally, two harbor seals (*Phoca vitulina geronimensis*) living in close proximity to one another presented with leukaemia of suspected viral aetiology (Griner, 1971).

##### (v) *Walrus*

We found reports on Atlantic walrus (*Odobenus rosmarus rosmarus*) and Pacific walrus (*Odobenus rosmarus divergens*). The only neoplasms reported in the Atlantic walrus were an osteosarcoma and a myelogenous leukaemia (Newman & Smith, 2006). An examination of 107 carcasses of Pacific walruses found 18 neoplasms, including leiomyomas, gastrointestinal tumours and hepatic tumours (Fleetwood, Lipscomb & Garlich‐Miller, 2005).

##### (vi) *Sea otters*

Neoplasia incidence in northern sea otters (*Enhydra lutris kenyoni*) has been estimated at 1–1.8% (Stetzer, Williams & Nightingale, 1981). There are a few reports on this species, among which are leiomyomas, lymphomas, seminomas and soft tissue sarcomas (Stetzer *et al.,* 1981; Reimer & Lipscomb, 1998; Kim *et al.,* 2002; Burek‐Huntington *et al.,* 2012).

In 2013, a study reported the first case of T‐cell lymphoma in sea otters. When affecting humans, this type of cancer is caused by a virus and usually occurs in immunosuppressed individuals. In the sea otter, however, no virus was found, and the pathogenesis remains to be clarified (Tanaka *et al.,* 2013).

##### (vii) *Cats*

A study conducted by Dorn *et al*. (1968) with reports from Alameda County, California, USA, estimated that 155.8 in 100000 cats developed cancer annually, compared to 381.2 in 100000 dogs. The reasons for the differences in cancer incidence between dogs and cats are unknown. Nevertheless, it seems that neoplasia could be as common in cats as it is in humans. The most common tumours in cats are lymphomas, most of which are caused by the feline leukaemia virus, which promotes the development of cancer by different means than that of human T‐cell lymphotrophic virus type 1 (Vail & MacEwen, 2000; Matsuoka, 2003).

Zambelli (2015) investigated the prevalence of feline cancer in South Africa and found 100 cases in a sample of 12893 cats. The most common cancer in this population was squamous cell carcinoma (48 of 100), which was three times higher in white/part‐white cats than in non‐white cats. This could be analogous to the higher susceptibility of light‐skinned humans to skin cancer. Curiously, melanomas, which are common in humans and dogs, are rare in cats (Cekanova & Rathore, 2014). Bladder cancer and osteosarcoma are two other tumours that are common both in humans and dogs, but rare in cats (Cekanova & Rathore, 2014).

The third most common type of tumour in cats is mammary cancers. Their architectural patterns are similar to those of humans, and the tumours are usually of the simple glandular type, which makes them histologically more similar to human mammary carcinomas than are dog carcinomas (Mulas & Reymundo, 2000). On the other hand, cat tumours are less hormone dependent than those of humans and dogs (Vail & MacEwen, 2000).

#### (i) *Order Chiroptera*

##### (i) *Bats*

Bats are the mammals with the highest lifespan to body mass ratio (Bourliere, 1958; Austad & Fischer, 1991), which Wilkinson & South (2002) suggested could be related to the decreased caloric intake of these animals during hibernation.

The longevity record among bats (41 years in a male) is held by the Brandt's bat (*Myositis brandtii*) (Podlutsky *et al.,* 2005). Researchers have discovered mutations in the growth hormone receptor (*GHR*) and in the insulin‐like growth factor 1 receptor genes (*IGF1R*) in these bats, both of which may contribute to their longevity (Seim *et al.,* 2013). GH and IGF1 both play roles in cell growth and proliferation, and these mutations could also affect the rate of cancer in these species. Additionally, Seim *et al*. (2013) found that the Brandt's bat, as well as another bat of the *Myositis* genus (*Myositis lucifugus*) have additional copies of the gene *FBX031*, which codes for a tumour‐suppressor protein. There are few reports of cancer in bats (Beck, Beck & Howard, 1982; Olds *et al.,* 2015), and none in the Brandt's bat.

#### (j) *Order Eulipotyphla*

##### (i) *African pygmy hedgehogs*

A study with 14 captive African hedgehogs (*Atelerix albiventris*) reported a cancer incidence of 30% (Raymond, White & Janovitz, 1997); another study with 66 hedgehogs reported an incidence of 53% with 85% of tumours classified as malignant (Raymond & Garner, 2001). Furthermore, about 10% of the hedgehogs had multiple types of tumours (Greenacre, 2004).

The mean age of individuals who developed the disease was 3.5 years (their maximum lifespan in captivity is approximately 11.4 years) (Greenacre, 2004; Tacutu *et al.,* 2013). In these studies, gender had no impact on cancer incidence. The most common tumours were malignant mammary adenocarcinoma, followed by lymphosarcoma and oral squamous cell carcinoma (Greenacre, 2004).

#### (k) *Order Proboscidea*

##### (i) *Elephants*

Like bowhead whales, elephants are large, long‐living animals that seem to resist the development of cancer. The cancer mortality among captive elephants (4.8%) is lower than that of humans (11–25%) (Abegglen *et al.,* 2015). This increased resistance could involve changes in tumour‐suppressor capacity. Abegglen *et al*. (2015) showed that elephants have 40 *TP53* alleles (humans have only two), leading to enhanced *TP53*‐mediated apoptosis. This gene is associated with the evolution of increased body size and enhanced response to DNA damage in elephants (Sulak *et al.,* 2016).

Elephants are one of the few animals whose reproductive fitness increases with age, which may have selected for their effective cancer‐suppression mechanisms (Poole, 1989; Abegglen *et al.,* 2015).

#### (l) *Order Sirenia*

##### (i) *Manatees*

Reports of neoplasia in manatees (*Trichechus manatus latirostris*) show cutaneous papillomas affecting various regions (Bossart *et al.,* 2002). Electron microscopy revealed papillomavirus particles within these tumours.

### (2) Marsupials and monotremes (non‐placental mammals)

The non‐placental mammals are the monotremes and the marsupials; we found reports on cancer in marsupials, but not in monotremes. Canfield, Hartley & Reddacliff (1990) catalogued all spontaneous proliferations in dasyurids (order Dasyuromorphia) and bandicoots (order Peramelemorphia) held by the Comparative Pathology Registry at Taronga Zoo. There were 70 proliferations among dasyurids and three among bandicoots. Among the common tumours in dasyurids were those of the adrenals, liver, and lymphatic system.

Isolated reports in sugar gliders (*Petaurus breviceps*) show one case of hemangiosarcoma (Rivas, Pye & Papendick, 2014), one case of concurrent adrenocortical carcinoma and hepatocellular carcinoma (Lindemann *et al.,* 2016), and two cases of metastatic mammary carcinoma (Keller *et al.,* 2014; Churgin *et al.,* 2015).

### (3) Other vertebrates

The phylogenetic proximity of other mammals to humans makes them the most suitable models for comparison and thus the main focus of our review. Non‐mammalian species, however, are also susceptible to cancer and may provide important data. Furthermore, investigating neoplasia in more phylogenetically distant species may reveal clues on the more primitive mechanisms of tumour suppression and maintenance of multicellularity. See Table 2 for a summary of cancer types in these animals.

#### (a) *Birds*

A review comprising data from multiple studies found that neoplasia has been reported more frequently in captive than in free‐flying wild birds, and more frequently in pet birds than in wild birds (Filippich, 2004). Researchers suggested the higher incidence in pet birds could be due to ease of observation, extended lifespan, higher exposure to carcinogens, and inbreeding (Castro *et al.,* 2016). Dietary changes and improvements in cancer treatment have extended the lifespan of pet birds, along with the incidence of neoplasia observed by veterinarians.

A literary survey of 1539 cases of neoplasia in pet birds found that the most common tumours were of the integument (31.7%), the urinary system (25.1%), and the genital system (17.3%) (Filippich, 2004). In Amazon parrots (*Amazona* spp.), however, the most common tumours are lipomas, lymphosarcomas, and fibrosarcomas (Levine & Practice, 2003).

#### (b) *Reptilians*

A retrospective study in reptiles held at the Philadelphia Zoological Garden examined 3684 necropsy reports from 1901–2002 and found 86 cases of neoplasia, six of which occurred in six chelonians (1.2% incidence), 22 in 19 lizards (3.0% incidence) and 58 in 53 snakes (2.9%). The most commonly affected organ in necropsied lizards and snakes was the liver, but in live snakes the incidence of skin tumours surpassed those of liver (Sykes & Trupkiewicz, 2006).

Compared with previous studies, the authors found an increase in incidence of neoplasia, which they suspected to be caused by increased longevity (due to improvements in husbandry and veterinary medicine), more thorough necropsy examinations, and by pathogenic infections (Sykes & Trupkiewicz, 2006).

Another retrospective study at the Sacramento Zoo analysed individual snake necropsies, medical records, and inventory records for the period 1 July 1981 to 30 June 1991. They found 20 snakes affected by 29 neoplasms, 19 of epithelial origin and 10 of mesenchymal origin (Ramsay *et al.,* 1996).

#### (c) *Amphibians*

Scientists have previously used amphibians as models to study cancer. For example, Schlumberger & Lucké (1948) used the leopard frog (*Rana pipiens*) to study the effect of temperature on cancer metastasis. Nevertheless, studies on both induced and spontaneous neoplasia are still lacking for this group.

Balls (1962) reviewed several reports of amphibian neoplasms and found a lower incidence of tumours for amphibians than for other species. This could be simply due to a lack of observations, but many authors have suggested mechanisms for tumour resistance, including one involving toad venom and another involving their potent regenerative ability. The author also mentions the early deaths of both captive and wild frogs as a possible explanation. Urodeles (salamanders) seem to have less spontaneous tumours than anurans (frogs), but this could have arisen from a lack of studies.

Tumours of the skin were the most common in amphibians. This could be explained by the easier observation of the skin compared to other organs, but the author suggests it could also be related to the high metabolic activity of the skin and to its constant contact with the environment, as opposed to visceral organs which are protected (Balls, 1962).

Tumours of the haematopoietic system are different in amphibians and fishes than in other animals. These two groups have no lymph nodes, and their main haematopoietic organs are the spleen, kidney, and intestinal submucosa instead of the bone marrow. Interestingly, the kidneys are the most common site of visceral tumours in amphibians (Balls, 1962).

#### (d) *Fishes*

Fishes are the largest class of vertebrates. Due to their economic importance, they are caught in large quantities and examined with care, leading to a greater quantity and quality of data on neoplasia in these animals (Schlumberger & Lucké, 1948). Emerging aging models like the turquoise killifish (*Nothobranchius furzeri*), a short‐lived vertebrate, also have a high incidence of cancer, mostly liver and kidney (Di Cicco *et al.,* 2011).

A review by Schlumberger & Lucké (1948) revealed that the most common benign epithelial tumours in fish are papillomas, many of which have an infectious aetiology. Adenocarcinomas, which are common in humans, were rare in these animals (Schlumberger & Lucké, 1948).

The most common neoplasms of the connective tissue were the benign and malignant tumours of the connective tissue proper, and the most common pigment cell tumours were melanomas (Schlumberger & Lucké, 1948).

Like amphibians, fishes do not possess lymph nodes and their haematopoietic organs are the spleen, mesonephros (the kidney), intestine, and gonads. 15 of the 20 reported lymphosarcomas in fish were present in the kidney (Schlumberger & Lucké, 1948).

Masahito, Ishikawa & Sugano (1988) point out that despite different organ morphology, many tumours in fish are histologically similar to those in humans, such as those of the liver and ovary. However, it is important to consider other biological features of these neoplasms, such as age. Wilms' tumour, for example, is a type of kidney cancer that in humans only develops in children and is often fatal, but in fish it may also affect adults.

Some fish tumours seem to have similar aetiology to human tumours. Researchers have isolated a herpesvirus in skin papillomas of salmonids, suggesting that viruses play a role in the development of cancer in fish. Hepatic tumours in fish are associated with polycyclic aromatic hydrocarbons, chemical contaminants that are implied in carcinogenesis in humans (Masahito *et al.,* 1988).

### (4) Invertebrates

Scientists previously thought only vertebrates could develop tumours. However, recent research supports the existence of tumours in invertebrates (Tascedda & Ottaviani, 2014).

Aktipis *et al*. (2015) searched for cancer in all lineages containing multicellular organisms and found cancer or cancer‐like disease in all but five lineages: Choanoflagellata, unicellular organisms that form simple multicellular colonies; Ctenophora and Placozoa, two groups of primitive organisms which lack many components involved in traditional neoplastic pathways; Porifera, which have no distinct tissues or organs, and some of which have a cell‐shedding mechanism used to remove damaged cells; and Hemicordata, which like elephants bear additional copies of *TP53*.

Research in cnidarians shows that even pre‐bilaterian animals can develop neoplasia (Domazet‐Lošo *et al.,* 2014). Two different species of *Hydra* have presented with tumour‐bearing polyps. The cells in these tumours were invasive, which suggests that metastatic capacity has ancient roots. Additionally, the tumours had several orthologues to tumour‐related genes in mammals and displayed parallels to the hallmarks of cancer in vertebrates. The proposed mechanism for tumourigenesis in *Hydra* is the accumulation of stem cells if they are not appropriately removed by programmed cell death (Domazet‐Lošo *et al.,* 2014). Scientists have also identified several cancer‐like lesions in corals (Aktipis *et al.,* 2015).

Flies of the *Drosophila* genus are among the best‐studied invertebrates. The most common tumours in this group are those of the lymph glands – which have hematopoietic function in *Drosophila –* and of haematocytes (Scharrer & Lochhead, 1950).

A haematopoietic tumour is known in molluscs; the cells responsible for these cancerous haematocytes overexpress heat shock protein 70 (*hsp70*), which codes for a protein that inactivates the tumour suppressor TP53 (Muttray *et al.,* 2010).

### (5) Plants

Like animals, plants also develop tumours. However, in plants the incidence of tumours is relatively low, and the tumours are not as lethal as those of animals (Doonan & Sablowski, 2010). Plant tumours can be caused by pathogens, and without pathogens most plants are not susceptible to neoplasia.

Plants also present with decreased rates of malignancy. One possible mechanism is that their cells are fixed in a cell wall matrix, which prevents the motility required to metastasise. This highlights the importance of interactions between a precancerous cell and the extracellular matrix in maintaining cell identity and preventing metastasis (Doonan & Sablowski, 2010).

Plants have orthologous genes of many mammalian tumour‐suppressor genes and oncogenes, but it seems that mutations in these genes are not oncogenic, possibly due to redundancy of some cell cycle regulators (Doonan & Sablowski, 2010).

## III. DISCUSSION

Our goal herein is to provide an overview of the diversity of cancer across the tree of life. Due to space limitations we cannot discuss in detail every report of cancer in all animal species, so we focus on summarizing the most important findings in mammals. A clear drawback was the lack of data for many species, and therefore our conclusions and interpretations are based on only a subset of species.

From an evolutionary perspective, the vulnerability that most animals have to cancer seems to be due to a combination of strong reproductive pressures and lack of selection for survival beyond reproductive age. For example, haematopoietic cancers are noteworthy for being common in almost all animals studied, and are common in humans during childhood and adolescence. This finding is in line with the idea that pressures exerted by pathogens on the immune system cause organisms to prioritize stronger defences at a cost of increased cancer vulnerability. Although scientists have already shown that infectious agents can induce tumour formation, we verify that their role in carcinogenesis extends to most living organisms, and we highlight studies in plants which show that pathogens are crucial for tumorigenesis in these organisms.

By contrast, a reduction in selection pressure against cancer causes cancer susceptibility to increase after reproductive age, which could explain the low incidence of cancer in wild animals, with their lower age expectancy. Elephants and whales are exceptions to this observation: they are some of the few animals that grow old in the wild. Elephants also increase in reproductive potential as they age. In such animals, selection for cancer resistance may persist, even for the elderly. Dogs, cats, humans, and mice, on the other hand, have become long lived because of the controlled environments they live in, and they are the mammals with the highest rates of cancer. One possible explanation could be the recent transition from the wild to the new environment, generating an evolutionary mismatch. Regardless of the reason, the extreme longevity of humans plays an important role in the high incidence of cancer in our species. We also note that the available data from animals in the wild may not be representative of their whole lifespan, since for many wild animals (e.g. cetaceans) we have a perspective of cancer focused on young individuals.

One striking finding from our work is that some human cancers are rare in non‐human primates. Prostate cancer is known to be rare in other species, which is not surprising because it is strongly age‐related. The few cases of testicular cancer in non‐human primates are harder to interpret because in humans, this cancer typically affects young men (Fig. 3), implying that we should be able to find it in other species (de Magalhães, 2013). It is possible that testicular cancer (like other cancer types) is strongly influenced by the human environment, as implied by different patterns in incidence in developed and developing countries (Ferlay *et al.,* 2013). Another possibility is that some post‐reproductive cancers are evolutionary accidents, random events in the evolution of species. Additional research comparing cancer incidence and cancer types in humans and non‐human primates is warranted.

The evidence discussed above suggests that the current rates of certain cancers in humans may be a product of an evolutionary mismatch with modern environments. The longer lifespans enjoyed in developed societies are also likely to be a major factor. Additionally, it is worth considering that cancer‐inducing genotypes occurring in less than 1/10000 may not be susceptible to negative selection, since this is the estimated limit of the resolution of natural selection for humans (Fisher, 1930).

Given the cancer resistance of humans with *GHR* deficiency (Guevara‐Aguirre *et al.,* 2011) and the cancer resistance of mice with multiple copies of *TP53* (García‐Cao *et al.,* 2002) or *INK4a/ADP ribosylation factor 1* (*ARF1*) (Matheu *et al.,* 2004), cancer resistance is probably not a hugely complex adaptation, even though there are certainly different evolutionary routes to achieve it. There are also possibly other adaptations in cancer‐resistant organisms, for example at the level of telomere length regulation (de Magalhães, 2013) or cell division timing (Maciak & Michalak, 2015). Indeed, we know very little about cell turnover in large mammals, including stem cells (Greaves & Ermini, 2015). Comparative studies of cell division rates, cell metabolism and cell turnover across mammals are a promising area of future inquiry. Lastly, short‐lived species may have a lower threshold for mutated cells to continue proliferating, given that their evolutionary priority is to grow quickly (Freitas & de Magalhães, 2011).

The absence of cancer in *GHR*‐deficient patients also suggests that cancer incidence may be fine‐tuned for each species: i.e. it is within a fairly narrow range described by longevity and body mass, even though the incidence of cancer can increase dramatically following single mutations. This fine‐tuning fits evolutionary theory in that cancer is largely age‐related, with a low incidence early in life. Another related observation is that the onset of cancer seems to occur in proportion to the species lifespan: there is a strong pressure to avoid cancer at earlier ages, which decreases at the end of reproduction.

## IV. CONCLUSIONS

(1) Cancer is pervasive across metazoans, and perhaps even across other kingdoms. We can assume that cancer affects all species of mammals.

(2) Although cancer may be unavoidable in animals with dividing cells that live long enough, data from elephants and bowhead whales suggest that it is possible to live for a long time with a large body mass, but have a low cancer risk. There is great variation in the age of onset and incidence of cancer across mammals, the genetic, cellular and molecular underpinnings of which are worthy of further inquiry.

(3) We do not yet know if the mechanisms identified in long‐lived, cancer‐resistant animals are responsible for their longevity and resistance to cancer, but some studies suggest that only a few mutations (or even a single mutation) can greatly increase cancer resistance.

(4) Our work highlights the different evolutionary pressures acting on cancer early in life (with a high prevalence of blood cancers presumably driven by the need to fight pathogens) and cancer late in life that escapes natural selection, including human‐specific cancers that may be evolutionary accidents or related to a mismatch with the modern environment.

(5) This review included published evidence from only a subset of species. It would be advantageous if researchers collected high‐quality evidence on cancer incidence in a wider range of animals, both in captivity and in the wild. As additional data become available, we will reach a better understanding of variation in cancer susceptibility and resistance in the extraordinary diversity of species on earth.
